# How It Affects the Nurses' Welfare

**Published:** 1919-12-06

**Authors:** 


### THE TRUE INWARDNESS OF STATE REGISTRATION.

### How it Affects the Nurses' Welfare.

So long as State Registration lay in the lap of the gods the nursing world was taught by a host of prophets that it was the one priceless treasure to be desired above all others. No matter from what affliction the nurse suffered, whether it was rheumatism or poverty, the tyranny of matrons, or the "charity" of the benevolent, these wiseacres contrived always to trace the cause to the lack of a State Register. The nurse was represented as the victim of a great national conspiracy to deny her her birthright. Now that Registration is in sight for England, Scotland, and Ireland, the symptoms of disillusion are making themselves manifest. It is becoming obvious that the trained nurse from Bart's or Guy's or St. Thomas's will gain nothing in status or privilege, or riches, by being permitted to describe herself as an "R.N." The seed that Dr. Addison has planted will indeed become a great tree, but many birds of doubtful plumage will be permitted to take refuge in its branches. Far-seeing people always recognise this as inevitable, and some of them, inspired by commendable jealousy for the status and reputation of those women who had sacrificed most in order to earn their diplomas, and sometimes to acquire other distinctions by their exertions and studies, opposed the one-portal system. State Registration was never discussed with judicial calm. It was made a war-cry, and a very large number of those who adopted it, and of those who ranged themselves amongst the opposition, knew little or nothing as to what it really signified.

### What Registration Stands for.

I am not at all sure that many people who think they understand really comprehend what State Registration stands for. It is generally looked upon as a sort of new machine, and the common attitude is to regard it with wonderment, and to wait to see what it will do. The Minister of Health is the chief necromancer, and he occupies the stage, presenting his marvellous invention to the gaze of the multitude. He has his claque and he has his critics—those who congratulate him on producing "non-contentious" Registration—after the factions had almost spilt blood in their endeavour to produce the same result—and likewise the doubting Thomases who regard the magician with "distrust and suspicion."

### An Essential Factor.

It is, I venture to suggest, of the utmost importance that nurses of all degrees should hold an intelligent view of State Registration and what it signifies. They should, moreover, understand the motive that lies behind the Act of Parliament, and the first lesson to learn is that *neither the Government nor the Minister of Health in introducing legislation is in the smallest degree concerned with the welfare of the nurse.* This may appear to be an astounding statement. It is probably one which many will contradict. I do not ask anybody to accept what I say without proper investigation. There have been many Registration Acts, among the first and greatest being the Medical Acts for the registration of medical practitioners. None of these Acts was passed for the benefit of the professional people concerned, but rather for the national benefit. I am quite sure from personal observation that the great majority of nurses think the Registration Act relating to them is a concession to the nursing profession. It is nothing of the kind. I go further than this, and, while I refrain from dogmatising, I hold the view that the only nurses who can hope directly to derive substantial benefit from the measure are the "birds of doubtful plumage" to whom I have referred—those who cannot show the hallmark of a first-class training. It may happen that the letters "R.N." after their names will make them eligible for public appointments from which they have been hitherto debarred. This remains to be seen. I do not wish to be misinterpreted or misunderstood. I neither affirm nor deny that the non-hallmarked R.N.'s who will find their names on the Register deserve recognition I am merely comparing pre-Act with post-Act conditions. And whether they deserve or receive benefit by the Act does not affect my statement that the motive which prompts this legislation is not the good of the nurse, but the good of the nation.

I particularly desire to convince the reader that this is true. It has never been appreciated that the nation owes more to the nurse than can ever be repaid. She is and has been a vital and indispensable factor in preserving the health of the nation. It may be true that in pursuing her vocation she is working for her livelihood. But, if so, the same is true of the soldier, the sailor, and the statesman. The nation has never recognised the value of the nurse's service, nor does this new Act, passed for the nation's benefit, recognise it. I hope that the College of Nursing will give this matter most serious attention. I am enunciating a principle of fundamental and far-reaching importance.

### The Motive Which Prompts.

No! The State Registration of Nurses is absolutely essential to the Minister of Health in the effective discharge of his duties, and this is the sole motive that prompts. It is as necessary that he should be able to measure the nursing strength of the country as that an Admiral should know to a pistol the strength of his fleet. The State Register will supply him with complete information. It will enable him to put his finger on the name, address, and qualifications of every working nurse in the country. It is right and necessary that this should be so, but it is equally right and necessary that the rank and file of the profession should realise that they are being registered so that they can be mobilised for national service, and not in order to provide butter or jam for their bread.

### An Obvious Corollary.

It was, indeed, never necessary to agitate for State Registration. It is the obvious corollary to a Ministry of Health, an essential part of the machinery. Nurses have been blindly clamouring for State Registration in the vain hope that in some mysterious way it might be the instrument of ministering to their "pressing necessities." They will soon be disillusioned. State Registration brings them no nearer to better pay, shorter hours, a pension on retirement, or any other material benefit. But indirectly it may serve their interests. It may demonstrate to the nation that they are public servants, not private employees, and that the high function which they fulfil deserves national and adequate recognition.

